# DHA Supplemented in Peptamen Diet Offers No Advantage in Pathways to Amyloidosis: Is It Time to Evaluate Composite Lipid Diet?

**DOI:** 10.1371/journal.pone.0024094

**Published:** 2011-09-08

**Authors:** Zareen Amtul, Mary Keet, Lin Wang, Peter Merrifield, David Westaway, Richard F. Rozmahel

**Affiliations:** 1 Department of Biochemistry, University of Western Ontario, London, Ontario, Canada; 2 Department of Anatomy & Cell Biology, University of Western Ontario, London, Ontario, Canada; 3 Lawson Health Research Institute, London, Ontario, Canada; 4 Centre for Prions and Protein Folding Diseases, University of Alberta, Edmonton, Alberta, Canada; Thomas Jefferson University, United States of America

## Abstract

Numerous reports have documented the beneficial effects of dietary docosahexaenoic acid (DHA) on beta-amyloid production and Alzheimer's disease (AD). However, none of these studies have examined and compared DHA, in combination with other dietary nutrients, for its effects on plaque pathogenesis. Potential interactions of DHA with other dietary nutrients and fatty acids are conventionally ignored. Here we investigated DHA with two dietary regimes; peptamen (pep+DHA) and low fat diet (low fat+DHA). Peptamen base liquid diet is a standard sole-source nutrition for patients with gastrointestinal dysfunction. Here we demonstrate that a robust AD transgenic mouse model shows an increased tendency to produce beta-amyloid peptides and amyloid plaques when fed a pep+DHA diet. The increase in beta-amyloid peptides was due to an elevated trend in the levels of beta-secretase amyloid precursor protein (APP) cleaving enzyme (BACE), the proteolytic C-terminal fragment beta of APP and reduced levels of insulin degrading enzyme that endoproteolyse beta-amyloid. On the contrary, TgCRND8 mice on low fat+DHA diet (based on an approximately 18% reduction of fat intake) ameliorate the production of abeta peptides and consequently amyloid plaques. Our work not only demonstrates that DHA when taken with peptamen may have a tendency to confer a detrimental affect on the amyloid plaque build up but also reinforces the importance of studying composite lipids or nutrients rather than single lipids or nutrients for their effects on pathways important to plaque development.

## Introduction

Gastric intolerance is one of the side effects of various treatments currently available for Alzheimer's disease (AD) [1]. Similarly, nutritional alterations and gastrointestinal dysfunction is common in various ailments including human immunodeficiency virus (HIV) infection (HIV) and so as in HIV associated dementia (HAD), which represents the most severe form of HIV-associated neurocognitive disorders [2]. HAD is commonly characterized by amyloid plaques deposition and other associated Alzheimer's disease (AD)-like brain pathology [3]–[7], resulting in neuronal loss with consequential dementia and death [8]. The amyloid plaques consist of different forms of the beta-amyloid (Aβ) derivatives of the amyloid precursor protein (APP) [9]. Through amyloidogenic pathway APP is first cleaved into C-terminal fragment β (CTFβ) by β-secretases, which is then cleaved by γ-secretase to produce Aβ40 and Aβ42 (reviewed in [Selkoe, 1998 #395]); the most common constituents of amyloid plaques. Where as non-amyloidogenic pathway initiated by α-secretases generates soluble APPα (sAPPα) and CTFα (reviewed in [Selkoe, 1998 #395]). Impaired functioning of proteins responsible for Aβ clearance such as insulin degrading enzyme (IDE) [10] is also accepted to aggravate AD pathogenesis, resulting in neuronal loss with consequential dementia and death [2].

Numerous studies in cell culture and animal models have traditionally evaluated the effects of dietary docosahexaenoic acid (DHA) alone; the most studied omega-3 polyunsaturated FA (PUFA) [11]–[15]. Diet usually consists of complex combinations of lipids or nutrients that might act synergistically or antagonistically and show a greater effect on disease than any single lipid or nutrient. The complexity of human diet, especially the high synergistic or antagonistic correlation among the effects of various nutrients and foods, makes it difficult to examine their separate effects [Willett, 1998 #2555]. In the current study, we investigated DHA with two dietary regimes; peptamen (pep+DHA) and low fat diet (low fat+DHA) for its effects on the pathological basis of AD in an AD mouse model. Peptamen is a standard sole-source nutrition for patients with gastrointestinal dysunction including HIV/AIDS [16] as well as Crohn's disease [17], stomach cancer [18], malignant gastroduodenal tumor [19], choledocholithiasis [20], acute pancreatitis [21], radiation enteritis and cystic fibrosis [22]. The testing of the potential effects of the combined use of peptamen diet supplemented with DHA therefore has important implications not only for AD but also for a significant number of patients suffering from HAD or long-term HIV survivors with co-morbid AD in the future [23]. Surprisingly, in contrast to low fat+DHA mice, TgCRND8 mice on pep+DHA diet showed an aggravated drift to produce beta-amyloid peptides and amyloid plaques. Our results suggest that beneficial effects of protective FAs (DHA) cannot be achieved perhaps without eliminating the deleterious fats from the diet and yet the amount of protective FAs (DHA) in the diet needs to be regulated.

## Materials and Methods

### Mice, treatment and tissue preparation

#### Ethics Statement

All experimental procedures were performed according to the animal care guidelines of the University of Western Ontario as approved by the University Committee on Research Ethics (UCRE; approval ID; 2004-065-06). All studies were performed on 5 month-old congenic C57BL/6J male mice heterozygous for transgene (**Tg**)CRND8 (which express a double-mutant form of human APP 695; Swedish (K670N/M671L) and Indiana (V717F) and demonstrate severe AD-like amyloid pathogenesis, including Aβ brain deposits as early as 3 months of age [24]. Mice used in these studies were housed on 12 h light/dark cycles. At 3 weeks of age TgCRND8 mice were provided *ad libitum* access to water and one of three diets (*n* = 6 for each diet) until 20–21 weeks of age at which time they were sacrificed by cervical decapitation. Brains were rapidly removed and sectioned sagitally into hemibrains. One hemibrain was used for the biochemical (protein, RNA and ELISAs) and lipid analyses. The other hemibrain was fixed in 10% neutral buffered formalin and 0.1 M phosphate buffered saline (PBS; 8.1 mM disodium hydrogen phosphate, 1.5 mM potassium dihydrogen phosphate, 137 mM sodium chloride, and 2.7 mM potassium chloride, pH 7.4) for 48 h and then stored at 4°C in PBS and 1% sodium azide until used for immunohistochemistry.

### Dietary manipulations

The experimental diet involved the following four groups: (**1**) Conventional mouse chow (**chow**; PicoLab Mouse Chow Diet 20; Purina Mills, St. Louis, MO) (**2**) Peptamen base nutrition diet (**pep**; Nestle Nutrition, Canada). Peptamen is a ready to use, isotonic, lactose-and gluten-free, whey peptide based isocaloric (1 kcal/ml) formula including 40 g of protein per 1000 kcal. Proteins are hydrolyzed into peptides (1% of free amino acids, 40% of peptides made of <10 amino acids, 46% of peptides of 10–40 amino acids, and 13% of peptides of >40 amino acids). Seventy percent of lipids are in the form of medium-chain triglycerides (11 g of long-chain triglycerides and 26 g of medium-chain triglycerides/1000 kcal) (**3**) peptamen base supplemented with 45.2 mg/20 ml/day/mouse of high level of algal origin DHA (**pep+DHA**; Neuromins, Pure Encapsulations, Inc., Sudbury, USA), (**4**) Soybean oil (1%), high protein, low fat diet (**low fat+DHA**; Research Diets Product L10047, New Brunswick). Composition of the low fat diet was as follows: alcohol extracted casein (304.7 g/kg), DL-methionine (4.6 g/kg), sucrose (279.3 g/kg), maltodextrin (Fro-Dex) (284.4 g/kg), vitamin Mix, V10001 (AIN-76A) (10.2 g/kg), mineral mix, S10001 (AIN-76A) (35.0 g/kg), cellulose (40.6 g/kg), xanthan gum (8.6 g/kg), soybean oil (10.2 g/kg) with DHA (Nu-Chek-Prep, Inc, USA). Low fat diet was supplemented separately by the recommended amount of essential linoleic (LA) and linolenic acids (LNA). In total, DHA made up roughly 5.76% of the total fat intake/mouse/day, which is almost half on a per weight basis to that of 13% omega-3 FA content of the high omega-3 diet of Greenland Eskimos, who have among the highest dietary intakes of omega-3 FA in the world [25]. In the present trial, we did not evaluate energy intake since the interventions were designed to be non-isocaloric. Special care was taken to make sure that the levels of vitamin E were identical across the diet groups.

### Fatty acid analysis

FAs in the brain were isolated and methylated according to Moser and Moser [26]. The FA methyl ester (FAME) mixture was then resuspended in hexane and analyzed by gas chromatography-mass spectroscopy (GC-MS). GC-MS analysis was performed on a Hewlett-Packard Series II 5890 gas chromatograph coupled to an HP-5971 mass spectrometer equipped with a Supelcowax SP-10 capillary column. FAME mass was determined by comparing areas of unknown FAMEs to that of a fixed concentration of 17:0 internal standard.

### Immunohistochemistry and image analysis

Plaque density was measured using immunohistochemistry with a biotinylated-4G8 monoclonal antibody (SIG-39240, Signet Laboratories, Dedham, MA), directed against amino acids 17–24 of the β-amyloid peptide. Briefly, brain tissue was fixed in 10% neutral buffered formalin, antigen retrieval performed with 70% formic acid (20 min., room temperature), endogenous mouse IgG was blocked using the MOM kit (Dako), followed by incubation with biotinylated-4G8 at a dilution of 1∶2000 and colour developed with DAB. Images were captured through an Olympus DP digital camera attached to an IX70 inverted microscope (Olympus) using Image Pro Plus Software. The images were analyzed using a macro to measure specifically stained area and total area. Plaques were examined (using 10× objective) in three brain regions (ten field each to cover the entire region): parietal cortex, hippocampus and thalamus: 0.5–0.9 mm, 0.9–1.35 mm, and 1.35–1.8 mm mediolateral from the bregma (Mouse Brain Atlas, Franklin and Paxinos 2007) on 40 µm thick rostral (cross) sections (6/region) of each hemisphere. Plaque density was calculated by dividing total area of Aβ-positive structures by per millimeter of each region analyzed (in square micrometers).

### Quantitative Western Blotting

Western blot to analyze proteins has been described previously [27]. Blots were bound to anti-APP (1∶15,000) from Sigma (A-8717), anti-β-site APP cleaving enzyme-C terminal fragment (BACE1-CTF) (1∶1000; a gift from Dr. Michael Willem), anti-β-actin (1∶5000) from Santa Cruz (sc-1616), anti-insulin degrading enzyme-1 (IDE-1) (1∶4000; a gift from Dr. Dennis Selkoe) and anti-prion protein (PrP) (1∶5000, a gift from Dr. Alex Strom). Goat anti-rabbit (RPN4301) was used at 1∶15,000 and sheep anti-mouse (RPN4201) at 1∶5000. Quantification was performed by using Image Gauge software with densitometry scans from Western blots.

### Real-time RT-PCR

RNA isolation, reverse transcription and PCR conditions have been described previously [27].The primers used are: TgAPP forward: 5′- GAT GAC GTC TTG GCC AAC ATG -3′ and reverse: 5′- CGG AAT TCT GCA TCC AGA TTC AC -3′ (308 bp product); murine APP: forward 5′- CCG ACG ATG TCT TGG CCA AC -3′ and reverse: 5′- CCG AAT TCT GCA TCC ATC TTC AC -3′ (310 bp product); PrP forward: 5′- GGG GAC AAC CTC ATG GTG GTA GT-3′ and reverse: 5′-TCC ACT GGC CTG TAG TAC ACT TGG-3′ (283 bp product); and β-actin forward: 5′-TCG TGG GCC GCT CTA GGC ACC A-3′ and reverse 5′- GTT GGC CTT AGG GTT CAG GGG GG-3′ (256 bp product). Data analysis was performed using the BioRad GeneX.

### Aβ sandwich ELISAs

Quantification of Aβ40 and 42 productions in brain was done by Signet ELISA methods. Briefly, parietal cortices of Tg mice were homogenized in 1 ml of 70% formic acid (to dissolve Aβ-positive aggregates) and centrifuged at 100,000×g for 1 hr. The supernatant was recovered and neutralized by a 20-fold dilution in 1 M Tris base buffer. Formic acid extracts of Aβ were diluted up to 1∶100 with diluents supplied with the kit (Signet Laboratories, Dedham, MA). Aβ40 and Aβ42 levels in the cortex were quantitatively measured by sandwich ELISA system using monoclonal antibodies (SIG-38940 and SIG-38942, Signet Laboratories, Dedham, MA). The Aβ values were calculated by comparison with a standard curve of synthetic Aβ40 and Aβ42 peptides, according to the protocol provided by the manufacturer.

### Statistics

All values were presented as mean ± standard error of the mean (S.E.M.). Protein densitometric and Aβ40 and Aβ42 levels and plaque density were compared by Student's unpaired two-tailed *t* tests. Ratios of CTFs to FA change were analyzed using one-way ANOVA analysis. The significance level was chosen to be 0.05 (*p*≤0.05). Correlations between parameters were tested by linear regression analysis.

## Results

### General health

All animals were routinely monitored for weight gain/loss (by weighing) and gastrointestinal problems (by monitoring feces and urine for texture and colour), agitation as well as sleep and general motor disorders. All animals were in good health with no obvious signs of any distress. No significant differences were observed in body weights between the Tg/chow and Tg/pep mice. Though Tg/pep+DHA mice were over 8% (27.7±3.37 vs 25.53±4.91, *p* = 0.0718) heavier than Tg/chow and Tg/pep mice, however, it did not reach to statistical significance. All animals were in good health with no obvious signs of disease.

### Effects on CNS lipid profile

GC-MS analysis of CNS lipids showed 6% increase in omega-3 FAs (DHA) in Tg/pep+DHA mice (1296.5±21.79 nmol/g, *p*<0.05) compared to Tg/pep (1221.92±18.66 nmol/g) mice. Where as Tg/low fat+DHA mice (1383.17±23.97 nmol/g, *p*<0.05) showed a 6.68% increase in brain DHA content compared to Tg/pep+DHA mice. Tg/low fat+DHA mice showed a 6.62% decrease in linoleic acid (99.79±3.929 nmol/g, *p*<0.05) compared to Tg/pep+DHA (106.86±2.81 nmol/g, *p*<0.05) mice, which had 7.72% lower linoleic acid compared to Tg/pep mice (115.8±1.199 nmol/g, *p*<0.05). The content of saturated fatty acid (SFA) was around 37.48% more in Tg/pep+DHA mice (16876.23±2070.32 nmol/g, *p*<0.05) compared to Tg/pep mice (12277.2±385.08 nmol/g, *p*<0.05). However, Tg/low fat+DHA mice (10149.42±790.33 nmol/g, *p*<0.05) had 39.8% lower SFA compared to Tg/pep+DHA mice. This data also indicates that 17±1 weeks is a reasonable time to alter CNS fatty acid concentration.

### Effects on plaque pathology

**Figure 1:**
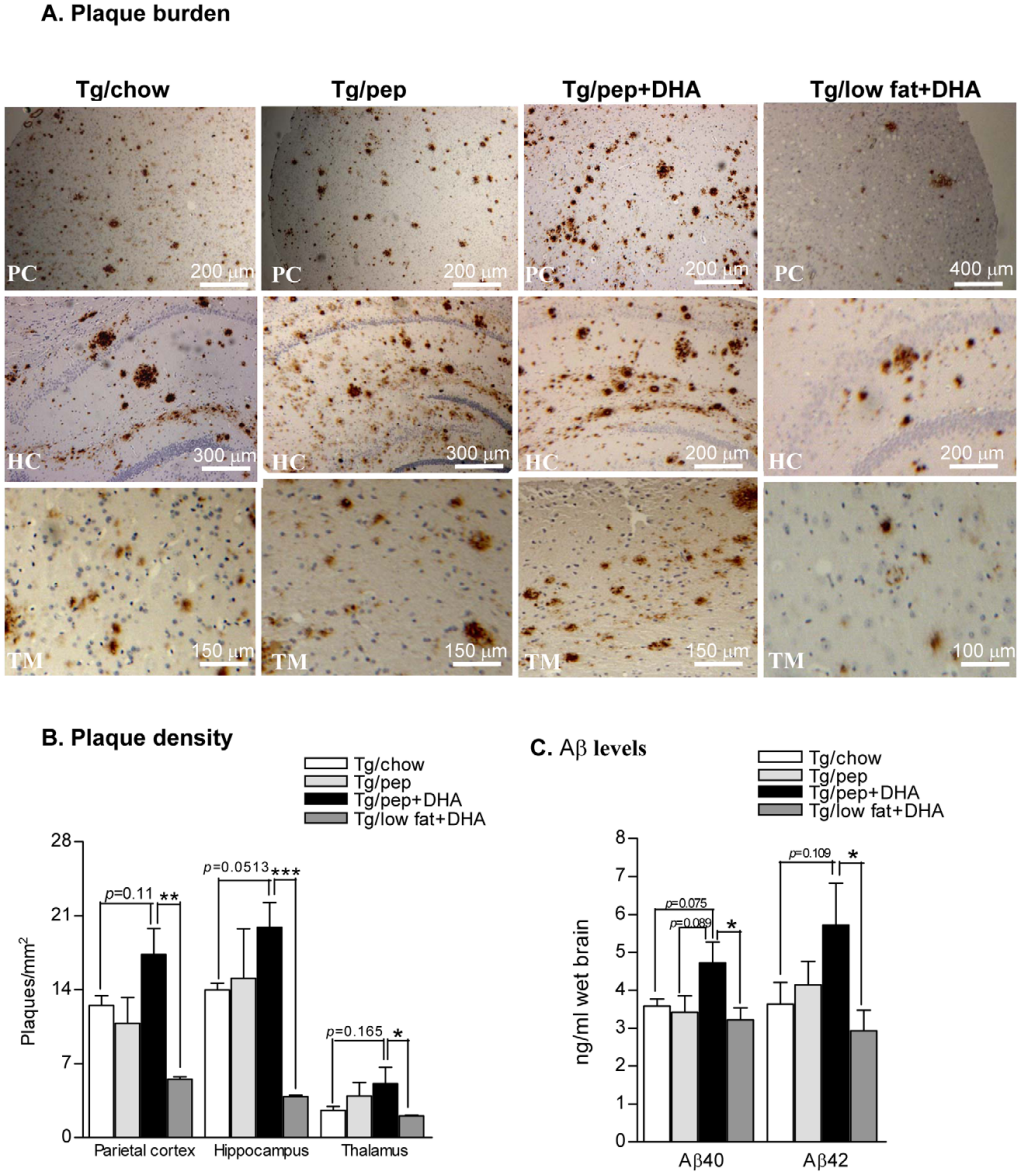
Effects of pep+DHA diet on amyloid plaques and Aβ levels. **A.** Photomicrographs of Aβ stained amyloid plaques in the Parietal Cortex (PC), Hippocampus (HC) and Thalamus (TM) of Tg/chow, Tg/pep and Tg/pep+DHA mice. **B.** Plot shows quantitative analysis of amyloid plaques density. **C.** ELISA results for Aβ40 and Aβ42 levels in the hippocampus and adjacent cortices of Tg/chow, Tg/pep, Tg/pep+DHA and Tg/low fat+DHA mice. The mean ± S.E.M. are shown for all plots, (n = 6 for each experiment), **p<0.01 and *p<0.05.

Immunohistochemistry and image analysis showed that pep+DHA supplementation, though non-significant, showed a detrimental trend on histopathological changes specifically that of 38% increase in amyloid plaque density in the parietal cortex (17.36±2.47 vs 12.53±0.912, *p* = 0.11), 42% in hippocampus (19.91±2.35 vs 13.98±0.658, *p* = 0.051) and 96% in thalamic (5.12±1.56 vs 2.61±0.34, *p* = 0.16) regions of Tg/pep+DHA mice brains when compared to Tg/chow mice brains, respectively (**Fig. 1A & B**).

Assuming that perhaps DHA and pep is not an ideal combination to decrease amyloid pathology in TgCRND8 mice, we designed “low-fat+DHA” diet, which was high in protein and DHA and low in all deleterious fats to find out if DHA is still detrimental. As expected, TgCRND8 mice on low fat+DHA diet showed a significant decrease in the plaque density/mm^2^ of parietal cortex: 5.5±0.19; *p*<0.01, hippocampus: 3.8±0.15; *p*<0.001, and thalamus: 2.0±0.62; *p*<0.05 compared to pep+DHA mice (**Fig. 1A & B**).

### Effects on Aβ levels

Levels of cerebral Aβ peptides were examined by ELISA, which is a standard method of detecting brain Aβ peptides in transgenic mice. In agreement with its effect on plaque burden, pep+DHA supplementation resulted in 32% increase in Aβ40 levels (4.73±0.55 vs 3.58±0.18 ng/ml, *p* = 0.075) and 57% increase in Aβ42 (5.72±1.09 vs 3.63±0.57 ng/ml, *p* = 0.109) levels in the Tg/pep+DHA mice brains compared to Tg/chow mice (**Fig. 1C**). Aβ40 was significantly (37%) lower in Tg/pep mice (3.43±0.43 ng/ml, *p* = 0.089) compared to Tg/pep+DHA mice. As expected, hippocampus and adjacent cortices of mice on low-fat+DHA diet demonstrated a significant 97% decrease in the levels of Aβ42 peptides (2.9±0.53; ng/ml; *p*<0.05) compared to pep+DHA mice (**Fig. 1C**).

### Effects on α-secretase pathway

**Figure 2:**
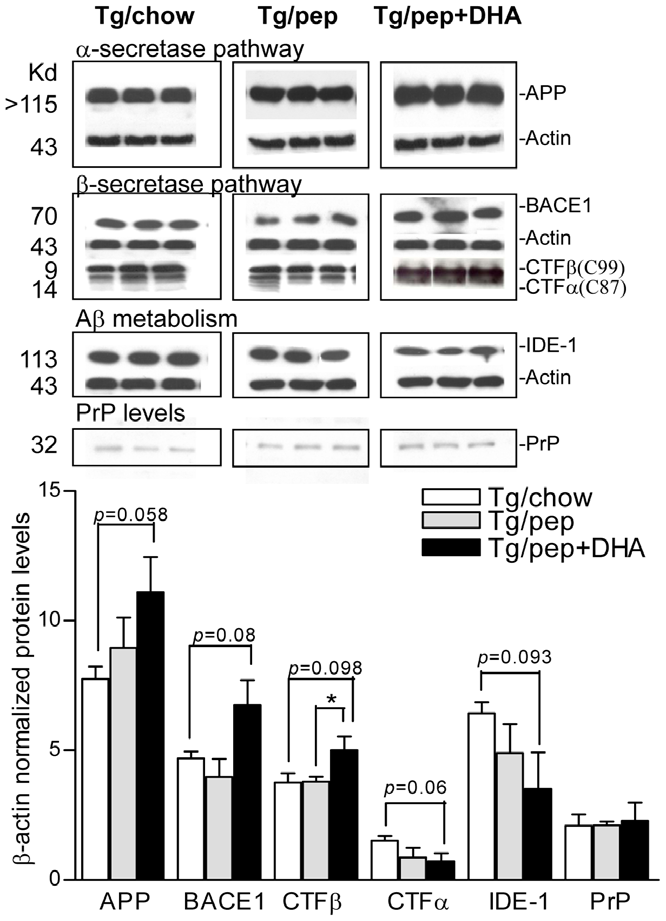
Effects of pep+DHA diet on protein levels. **A.** Western blots of APP, BACE, APP-CTFs (α/β), IDE, PrP and β-actin protein levels in the frontal cortices of Tg/chow, Tg/pep and Tg/pep+DHA mice. Respective molecular weights (Kd) are shown on the left. Plot shows quantitative analysis of protein levels as mean ± S.E.M., (n = 6 for each experiment, out of 6 only 3 animals each are shown for Tg/chow, Tg/pep and Tg/pep+DHA mice in Western blots), ***p<0.001, **p<0.01 and *p<0.05.

Western blot showed 15% increase in the levels of membrane bound FL-APP (8.95±1.16 vs 7.75±0.47, *p* = 0.058) and 47% decrease in the levels of APP-CTFα (0.72±0.30 vs 1.52±0.17, *p* = 0.06) in the frontal cortices of Tg/pep+DHA mice compared to Tg/chow mice, respectively (**Fig. 2A**).

### Effects on β-secretase pathway

Western blot showed 43% increase in the levels of BACE (6.73±0.96 vs 4.69±0.26, *p* = 0.08) with the corresponding 33% increase in the levels of APP-CTFβ (5.0±0.52 vs 3.75±0.36, *p* = 0.098) in Tg/pep+DHA mice compared to Tg/chow mice, respectively (**Fig. 2A**). Though there was no significant difference in BACE levels between Tg/pep+DHA and Tg+pep mice, however, CTFβ levels were significantly (*p*<0.05) lower in Tg/pep mice compared to Tg/pep+DHA mice.

### Effects on Aβ metabolism

To determine the relatively larger increases in Aβ levels compared to the smaller increases in APP-CTFβ, we next examined the levels of IDE (proteins responsible for the clearance of Aβ). Western blot showed 54% reduction in the IDE levels (3.51±1.40 vs 6.43±0.42, *p* = 0.093) in Tg/pep+DHA mice compared to Tg/chow mice, respectively (**Fig. 2A**).

### Effects on PrP levels

The CRND8 transgenic APP expression is driven by the PrP promoter. To support the absence of the effect of different diets on PrP activity - the protein levels of PrP among different groups were investigated. As shown in **Fig. 2A**, the different dietary regimens did not cause any significant change in PrP levels, thereby supporting that both the pep and pep+DHA diets did not influence the levels of APP transgene expression.

### Effects on secretase products

**Figure 3:**
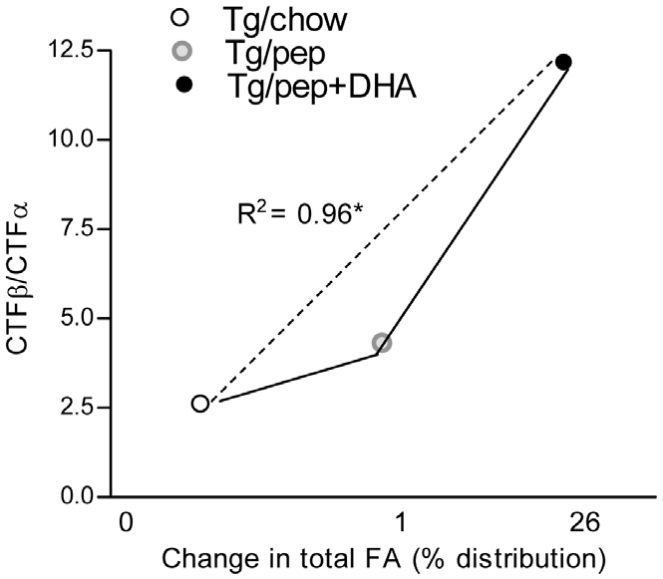
Effects of pep+DHA diet on α/β- secretase products. Change in percentage distribution of total FA in the CNS (**Table-1**) was plotted against CTFβ/CTFα ratio (**Figure 2**) of Tg/chow, Tg/pep and Tg/pep+DHA mice. Plot shows a decrease in CTFβ/CTFα ratio with increasing FA percentage distribution, *p<0.05. The mean ± S.E.M. is shown for, *p<0.05.

Significant direct correlation (R^2^ = 0.96; *p*<0.05) was found between increasing percentage distribution of CNS FAs versus the ratios of the levels of secretase products; CTFβ (generated by β-secretase) and CTFα (generated by α-secretase) (**Fig. 3**). This correlation suggests that the activities of α and β-secretase enzymes are affected by the CNS FAs levels. This influence of FA levels on CTFβ/CTFα ratio and consequently on Aβ production can be partly rationalized in that APP folding, directed trafficking to proteosomal degradation or post-translational processing, as well as cleavage by the secretases occurs within the milieu of specific cellular membranes, and that their lipid architecture can affect such activity and/or specificity.

### Effects on endogenous APP, transgene and PrP gene expressions

**Figure 4:**
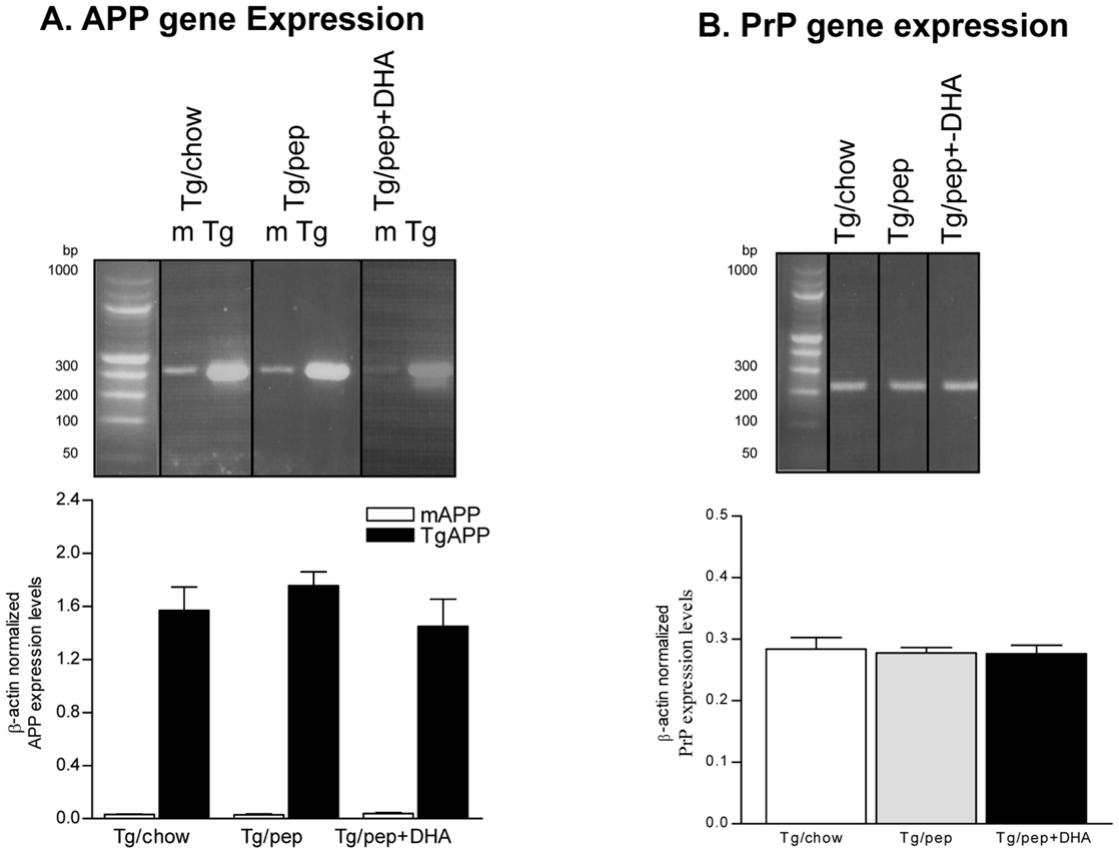
Effects of pep+DHA diet on gene expression levels. Real-time RT-PCR of endogenous murine (m), CRND8 transgene-specific (Tg) APP (**A**) and PrP (**B**) expression levels in Tg/chow, Tg/pep and Tg/pep+DHA mice, (n = 6 for each experiment, out of 6 only 1 animal is shown for each group in PCR images). Plots show quantitative analysis (mean ± S.E.M.) of β-actin normalized gene expression levels.

In order to investigate if pep and pep+DHA-induced increase in APP levels is due to the altered transcription of APP gene (or PrP gene; that derives the transcription of APP in TgCRND8 mice), real-time RT-PCR was carried out. **Fig. 4A** showed that both the pep and pep+DHA diets did not have any effect on the expression levels of either CRND8 transgene-specific APP or endogenous APP (**Fig. 4A**). Similar to PrP protein levels, the levels of PrP gene were comparable across the groups (**Fig. 4B**).

## Discussion

In this study, we reinforce our recent findings [28]–[31] that physiological interaction among amalgamated mixtures of different FAs and/or nutrients in a diet, is critical for their impact on Aβ production/metabolism. Attributing the effects of a composite diet to a single lipid alone may not be adequate to explain most of the effects of that particular diet. Instead, it is important to investigate as well as discuss the optimal proportion of lipids/nutrients that are frequently or seldom present in a diet and might offer the most favorable biological synergy in relation to disease risk. For instance, DHA demonstrates an overall drift towards detrimental consequences - when it is administered with peptamen based liquid - in pathways important to amyloid deposition. This drift appears to be the result of a combination of several contributing factors. First, in Tg/pep+DHA mice proteolytic processing of the membrane-bound mature FL-APP was roughly augmented leading to corresponding increases in APP-CTFβ as compared to their age-matched Tg/pep and Tg/chow controls. Since the expression levels of the APP transgene and PrP promoter were unchanged, the elevated trend in FL-APP protein is most likely the result of its diminished degradation through the proteasomal pathway [3] or its increased overall stability, implying that pep+DHA diet may alter Aβ generation either by altering APP trafficking to secretase-containing compartments of the membrane or secretase enzymatic activity itself. In keeping with the proposed diminishment of degradation - precedence for the impact of differential FAs on activity of the ubiquitin-proteasome pathway is seen with other proteins [32], suggesting a physiological means to regulate protein degradation where post-ER trafficking to either the Golgi or proteosomes appears the underlying basis.

Secondly, the increase inclination in BACE levels is likely due to the elevation of its FL-APP substrate - in agreement with a study demonstrating a parallel increase in BACE with levels of FL-APP [33]. Alternatively, since BACE is also degraded via the ubiquitin-proteasome pathway [34] – the affect of pep+DHA supplement on BACE levels may also be the result of diminished proteosomal degradation, or a combination of the two. Nevertheless, given the complexity of the *in vivo* system the actual basis of this effect would be difficult if not impossible to ascertain.

Thirdly, and of particular significance, in Tg/pep+DHA mice - pep+DHA enrichment resulted in a leaning towards augmentation in Aβ levels with 57% increase in Aβ42 levels alone, more than would be expected by elevation of BACE and CTFβ alone. Consequently, the IDE levels were 54% reduced. IDE, which appears to degrade intracellular Aβ more effectively than does neprilysin in both the detergent-soluble and insoluble fractions [35].

Lastly, knowing that DHA and the brain are rich in oxidizable PUFA we propose that perhaps (conversely to low fat+DHA diet) some oxidizable constituents in peptamen diet might have caused lipid membrane peroxidation and/or free radical damage to the TgCRND8 mice brains [36] and antagonized the beneficial effects of DHA either by increasing 4-hydroxynonenal (4-HNE), which also reports to activate BACE indirectly [4], as observed in this study or due to impaired Aβ clearance, by somehow, reducing the expression of the gene coding for IDE. Further *in vitro* studies in neuronal cell lines would be required to clarify the basis of these effects.

To evaluate if DHA has protective effect when supplemented with a different composite nutritional base, we designed a diet high in DHA but low in all deleterious associations with fats, and showed that DHA indeed improve AD-type neuropathology in the brains of TgCRND8 mice. Initially, by significantly decreasing hippocampal Aβ40 and Aβ42 peptides levels and subsequently by reducing amyloid plaque burden in cortex, hippocampus as well as thalamus, endorsing our recent observation [31]. Though the depletion of deleterious fat alone from the diet has shown reduced amyloidosis [31], however, DHA supplementation even enhances this protective effect further.

Peptamen is largely composed of ready to digest saturated fatty acids, which apparently perhaps the reason for ∼37% increase in saturated fatty acids in Tg/pep+DHA mice. Tg/pep mice despite having lowest omega-3 (DHA) levels among all mice groups did not show as adverse plaque pathology as Tg/pep+DHA mice (with significantly higher DHA levels than Tg/pep mice). Though Tg/pep mice also showed an increase in saturated fatty acids, however, not as huge as in Tg/pep+DHA mice, perhaps supposedly due to the altered metabolism of saturated fatty acids and DHA combination or due to the toxic interaction(s) between DHA and it's metabolites (like docosanoids) and saturated fatty acids. Since the effects of such interactions are not fully understood, the possibility that they may have adverse affects on neuronal survival cannot be excluded. Linoleic acid deficiency reflects a decrease in the omega-6 fatty acids as DHA has been shown to down-regulate arachidonic acid (a member of omega-6) pathway [37]. Therefore, higher levels of DHA were found to be associated with lower levels of linoleic acid in the present study. Moreover, a 2–9% increase in CNS DHA in the present study is reasonable for 4–5 months treatment, because other studies report that large (50–80%) [38] alteration in brain DHA requires multiple generations of animals on DHA-depleted diets [39].

Our results suggest that perhaps the beneficial effects of DHA can not be seen if they are supplemented without taking the effect of other blended nutrients into consideration, especially if that nutrient affects DHA antagonistically. Our findings also explain reported controversial roles (detrimental or no effects) of diets rich in omega-3 FAs on AD pathology. For instance, high omega-3 diet was unable to improve cognitive performance in Tg mice in multiple cognitive measures [15], [40]. Further to this, the administration of DHA, EPA or placebo to a group of mild to moderate AD subjects for 6 months resulted in no difference in mini mental state examination (MMSE) or AD Assessment Scale (ADAS) scores between the groups, despite daily omega-3 doses that were many times higher than intake of omega-3 FA in fish products [41]. Similarly, in non-transgenic rodents, there was no cognitive benefit to supplementation with DHA after multiple generations of omega-3 deficiency [42], [43]. Likewise, Calon et al., reported no benefit in recognition/identification memory or memory retention in either aged non-transgenic or aged Tg2576 mice supplemented with DHA following depletion [13]. In the same way, MIDAS [44] and ADCS [45] DHA trials did not show a benefit of DHA treatment in mild to moderate AD. Similarly an increase in neuronal loss and prion formation [46] in cell-culture AD models after DHA supplementation has also been reported. Ironically, none of these studies had taken into account the role and effect of other lipids or nutrients in the diet, that may had either complemented or antagonized the effects of DHA on the risk of AD.

In summary, our results from *in vivo* mouse studies opened up new alleys in understanding the complex roles of PUFAs on the biochemical mechanisms leading to amyloid plaque development in patients suffering from co-morbidities. Present data do not argue the protective effects of DHA (as shown by low fat+DHA diet), rather strengthen ours [28]–[31], [47] and others [48] previous findings that we may have to focus on diet mixtures rather than associating all effects to a single lipid or ingredient. We assume that cellular/molecular homeostasis mechanisms can effectively offset the effects of single lipids, rather than complex synergistic or antagonistic changes exerted by mixture of diets. Further controlled *in vitro* investigations, and particularly long-term human-based retrospective studies would be required to confirm the effect of pep+DHA diet and justify dietary modifications for patients at risk of suffering from AD and HAD like co-morbidities.
