# Correction: DHA Supplemented in Peptamen Diet Offers No Advantage in Pathways to Amyloidosis: Is It Time to Evaluate Composite Lipid Diet?

**DOI:** 10.1371/journal.pone.0176644

**Published:** 2017-04-20

**Authors:** Zareen Amtul, Mary Keet, Lin Wang, Peter Merrifield, David Westaway, Richard F. Rozmahel

Fig 2 is incorrect. The appearance of certain bands on Western blot images are compromised. The actin bands of Tg/pep in b-secretase pathway section are switched with the actin bands in Tg/pep+DHA bands of Abeta metabolism section. The authors have provided a corrected version here.

**Fig 2:**
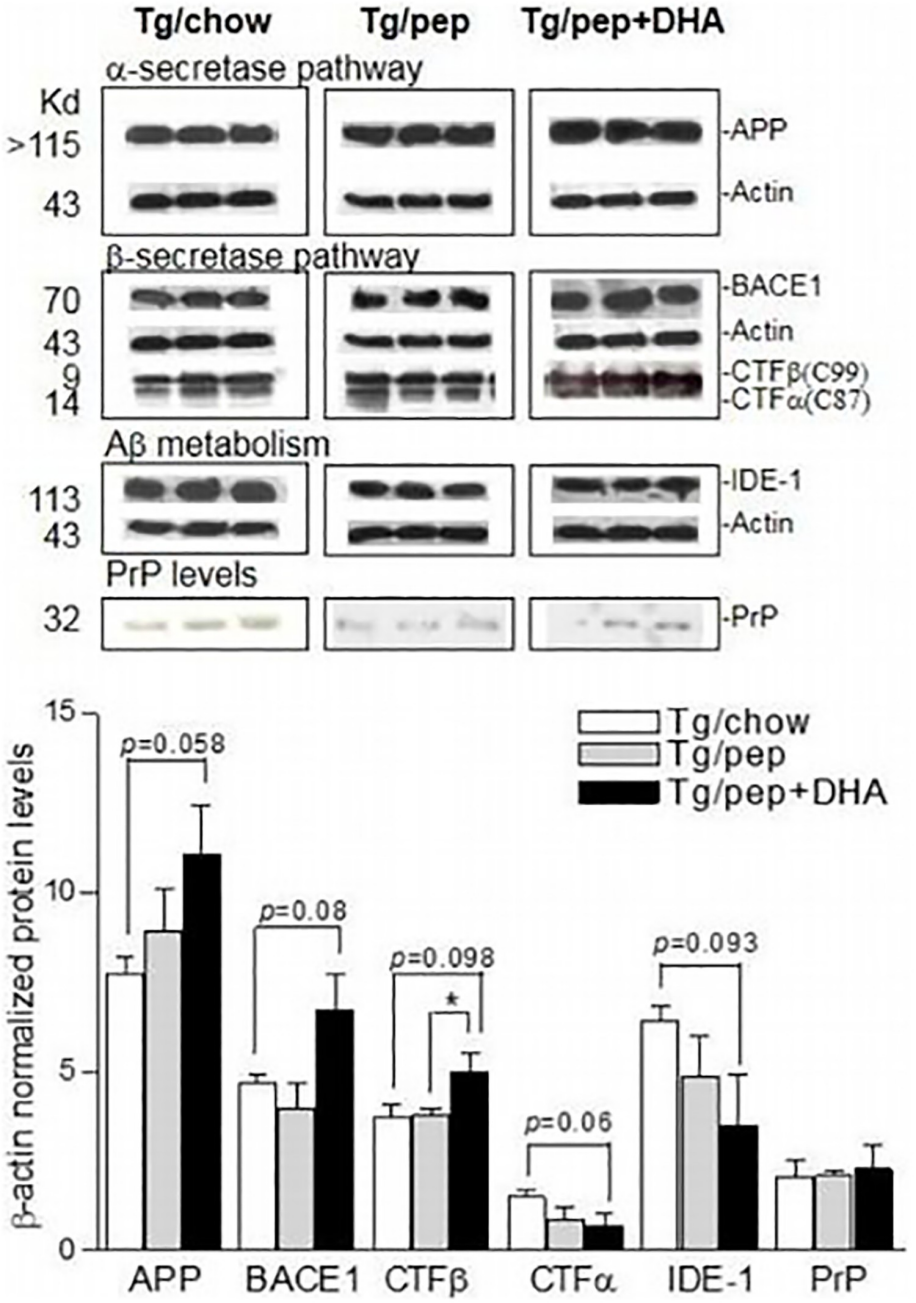
Effects of pep+DHA diet on protein levels. A. Western blots of APP, BACE, APP-CTFs (α/β), IDE, PrP and β-actin protein levels in the frontal cortices of Tg/chow, Tg/pep and Tg/pep+DHA mice. Respective molecular weights (Kd) are shown on the left. Plot shows quantitative analysis of protein levels as mean ± S.E.M., (n = 6 for each experiment, out of 6 only 3 animals each are shown for Tg/chow, Tg/pep and Tg/pep+DHA mice in Western blots), ***p<0.001, **p<0.01 and *p<0.05.
